# A Novel Methodology: the Fixed-Quality Variable-Type Dietary Intervention

**DOI:** 10.1016/j.advnut.2025.100508

**Published:** 2025-09-07

**Authors:** Andrew A Bremer

**Affiliations:** Office of Nutrition Research, National Institutes of Health, Bethesda, MD, United States

How many times has the comment “if only we had improved methodologies…” been uttered from nutrition science researchers attempting to address all the complexities involved with performing and interpreting dietary intervention studies. Well, now we can add one more to the armamentarium.

As described in their article, “Nutrition Research & Programming in Multicultural Populations: The Fixed-Quality Variable-Type (FQVT) Dietary Intervention” [1], Katz and Gardner introduce a novel paradigm that: *1)* addresses significant gaps in current clinical nutrition research methodologies and *2)* aims to enhance the applicability, adherence, and effectiveness of dietary interventions in diverse populations. Specifically, Katz and Gardner [1] propose an innovative approach to dietary intervention research that accommodates the multicultural diversity of study participants and population members. They suggest that the FQVT nutrition intervention—which standardizes diet quality using objective measures while allowing for a range of diet types that cater to individual preferences, tastes, upbringings, ethnicities, and cultures—has the potential to enhance participant satisfaction, adherence, and long-term maintenance of dietary practices while also maintaining internal validity and enhancing external validity of dietary interventions. As such, they further suggest that the FQVT intervention has broad applications beyond research settings and the potential to improve the effectiveness of food is medicine (FIM) and other diet-based approaches to personal and public health.

Importantly, Katz and Gardner [1] highlight several issues that are relevant for enhancing the rigor and generalizability of dietary intervention studies:

• Accommodating diverse dietary preferences, tastes, and cultural backgrounds. Dietary intervention studies typically utilize a 1-size-fits-all approach. Although convenient, this approach does not account for the cultural and personal preferences of study participants and may unfortunately compromise adherence and diminish the generalizability of study findings. However, as the authors describe, the FQVT approach to dietary intervention studies allows for the inclusion of diverse dietary patterns that reflect the cultural and ethnic background of study participants; this is particularly relevant in multicultural societies (like the United States) where dietary habits vary widely. By accommodating these differences, the FQVT approach enhances the cultural relevance and acceptability of dietary interventions and has the potential to enhance participant satisfaction and adherence. The authors suggest that this may thereby improve the overall effectiveness of dietary interventions and make them more applicable in real-world settings across diverse populations [2,3].
• Standardizing and objectively measuring diet quality. The FQVT method standardizes the objective measure of diet quality using validated tools such as the Healthy Eating Index (HEI) 2020 [4]. Standardizing diet quality ensures that all intervention diets, regardless of type, meet a prespecified level of nutritional optimality. The authors highlight how this approach allows for a fair comparison of different dietary patterns while maintaining the integrity of nutritional goals; it also shifts the focus from specific diet types to overall diet quality, which is consistently linked to better health outcomes [5]. Importantly, the use of the HEI-2020 as a benchmark for diet quality is well-supported by existing literature, demonstrating its robustness and reliability [6,7]. A recent adaptation of the HEI also potentially makes it an even more useful measure for FQVT studies by tailoring it to address the variations in discretionary food-group intake in multicultural populations [8]. Importantly, the FQVT approach further has the potential to end long-running debates about which type of diet is better—for example, a low-carbohydrate diet or a low-fat diet. Investigators have traditionally compared these types of diets head-to-head, and results have frequently been inconsistent due to myriad factors including study design. But, with the FQVT approach, diet quality is matched and fixed; so, in fact, a low-carbohydrate diet and a low-fat diet (or any other diet for that matter) could be part of the same intervention. Only by matching diets for overall quality—and not conflating differences in quality with differences in composition—can we learn about the potential differences among dietary patterns and health outcomes.
• Providing flexibility and personalization in dietary patterns. The FQVT approach allows for a range of diet types tailored to individual preferences and tastes while maintaining fixed nutritional standards. By offering a plurality of dietary patterns that meet the same quality standards, the FQVT method allows for a wide range of dietary patterns, each tailored to the individual’s unique preferences while maintaining a standardized level of diet quality. As the authors discuss, this flexibility may lead to better adherence and sustained dietary changes because participants are more likely to continue diets that align with their personal preferences [9,10].

Katz and Gardner [1] also highlight in their article how the FQVT dietary intervention is relevant to personal and public health:

• Enhanced adherence and long-term maintenance. The authors suggest that the personalized nature of the FQVT method, combined with its focus on maintaining high diet quality, may improve not only short-term adherence to dietary interventions but also long-term adherence to dietary interventions. This will need to be validated in studies adopting this approach; but, it is plausible and extremely important given that adherence to dietary interventions is a critical factor in achieving desired health outcomes and sustained health improvements.
• Feasibility with existing and emerging tools. Given the multimodal nature of medicine and the utility of leveraging diverse methodologies in research and clinical practice, the authors highlight the feasibility of implementing the FQVT approach with existing and emerging dietary assessment tools, such as diet quality photonavigation and digital dietary assessment methods [11,12]. These tools enable rapid and accurate assessment of dietary intake and quality, making it feasible to implement the FQVT approach in both research and public health settings. Moreover, the integration of these tools significantly reduces the time and effort required for dietary assessment, facilitating large-scale implementation.
• Potential for broad application. By providing a framework that accommodates individual preferences while maintaining high diet quality, the FQVT approach may enhance the effectiveness of dietary interventions in various contexts. As such, the authors suggest that the FQVT methodology is relevant to FIM and other diet-based approaches to personal and public health, especially because it can be effectively integrated into clinical interventions and public health nutrition programs. For example, a specific component of the FIM paradigm is the integration of nutrition into health care through the prescription of medically tailored meal plans to manage and prevent chronic diseases [13]. The FQVT approach offers a structured yet flexible framework that can be readily adopted into such programs, ensuring that dietary interventions are both culturally appropriate and nutritionally sound [14].

In summary, the FQVT dietary intervention represents a novel advancement in nutrition research methodology. By prioritizing diet quality and accommodating individual and cultural preferences, the FQVT approach addresses key limitations of traditional dietary intervention studies. Its potential to enhance adherence, satisfaction, and external validity also makes it a promising tool for both research and public health applications. As the global nutrition landscape continues to evolve, the FQVT approach offers a versatile and promising framework for developing and implementing dietary interventions that are both scientifically robust and culturally relevant.

## Funding

The author reported no funding received for this study.

## Conflict of interest

AAB is an Editor for *Advances in Nutrition* and played no role in the Journal’s evaluation of the manuscript. The contents of this article represent the author’s views and do not constitute an official position of the National Institutes of Health or the United States Government.
